# Prehospital risk stratification in patients with chest pain

**DOI:** 10.1136/emermed-2020-210212

**Published:** 2021-08-09

**Authors:** Dennis Sagel, Pieter Jan Vlaar, Radboud van Roosmalen, Ingmar Waardenburg, Wybe Nieuwland, Roelof Lettinga, Robert van Barneveld, Edward Jorna, Roelof Kijlstra, Carien van Well, Antoon Oomen, Louis Bartels, Rutger Anthonio, Vincent Hagens, Sjoerd Hofma, Youlan Gu, Derk Drenth, Ryanne Addink, Thea van Asselt, Peter van der Meer, Eric Lipsic, Luis Juarez Orozco, Pim van der Harst

**Affiliations:** 1 Expirimental Cardiology, University Medical Center Groningen, Groningen, The Netherlands; 2 Department of Cardiology, Catharina Hospital, Eindhoven, The Netherlands; 3 Department of Cardiology, Wilhelmina Hospital Assen, Assen, The Netherlands; 4 Department of Cardiology, University Medical Center Groningen, University of Groningen, Groningen, The Netherlands; 5 Department of Anthesiology, University Medical Center Groningen, University of Groningen, Groningen, The Netherlands; 6 Department of Emergency Medical Services, University Medical Center Groningen, Groningen, The Netherlands; 7 Department of Anesthesiology, Hospital Nij Smellinghe, Drachten, The Netherlands; 8 Emergency Medical Services, Kijlstra ambulance zorg, Drachten, The Netherlands; 9 Cardiology, Antonius Hospital Sneek, Sneek, The Netherlands; 10 Department of Cardiology, Martini Ziekenhuis, Groningen, The Netherlands; 11 Cardiology, Treant Zorggroep Locatie Scheper, Emmen, The Netherlands; 12 Department of Cardiology, Ommelander Hospital Groningen, Scheemda, The Netherlands; 13 Department of Cardiology, Medical Center Leeuwarden, Leeuwarden, The Netherlands; 14 Department of Cardiology, Hospital Nij Smellinghe, Drachten, The Netherlands; 15 Avicenna General Practice Paterswolde, Paterswolde, The Netherlands; 16 Middelstum General Practice, Middelstum, The Netherlands; 17 Department of Epidemiolgy, University Medical Center Groningen, Groningen, The Netherlands

**Keywords:** cost effectiveness, emergency ambulance systems, effectiveness, cardiac care, acute coronary syndrome, prehospital care

## Abstract

**Objectives:**

The History, ECG, Age, Risk Factors and Troponin (HEART) Score is a decision support tool applied by physicians in the emergency department developed to risk stratify low-risk patients presenting with chest pain. We assessed the potential value of this tool in prehospital setting, when applied by emergency medical services (EMS), and derived and validated a tool adapted to the prehospital setting in order to determine if it could assist with decisions regarding conveyance to a hospital.

**Methods:**

**In 2017,** EMS personnel prospectively determined the HEART Score, including point-of-care (POC) troponin measurements, in patients presenting with chest pain, in the north of the Netherlands. The primary endpoint was a major adverse cardiac event (MACE), consisting of acute myocardial infarction or death, within 3 days. The components of the HEART Score were evaluated for their discriminatory value, cut-offs were calibrated for the prehospital setting and sex was substituted for cardiac risk factors to develop a prehospital HEART (preHEART) Score. This score was validated in an independent prospective cohort of 435 patients in 2018.

**Results:**

Among 1208 patients prospectively recruited in the first cohort, 123 patients (10.2%) developed a MACE. The HEART Score had a negative predictive value (NPV) of 98.4% (96.4–99.3), a positive predictive value (PPV) of 35.5% (31.8–39.3) and an area under the receiver operating characteristic curve (AUC) of 0.81 (0.78–0.85). The preHEART Score had an NPV of 99.3% (98.1–99.8), a PPV of 49.4% (42.0–56.9) and an AUC of 0.85 (0.82–0.88), outperforming the HEART Score or POC troponin measurements on their own. Similar results were found in a validation cohort.

**Conclusions:**

The HEART Score can be used in the prehospital setting to assist with conveyance decisions and choice of hospitals; however, the preHEART Score outperforms both the HEART Score and single POC troponin measurements when applied by EMS personnel in the prehospital setting.

**Key messages**

**What is already known about this subject**

- Although the History, ECG, Age, Risk Factors and Troponin (HEART) Score is frequently used in risk-stratifying patients, there is limited evidence regarding its ability to accurately risk stratify patient in the prehospital setting.
- Available reports predominantly reveal unacceptable rates of adverse events in patients stratified to low risk, and no initiative has generated or adapted a risk score to the prehospital evaluation of patients with chest pain.

**What this study adds**

- In this prospective study of patients conveyed by emergency medical services (EMS) with chest pain, we found that the HEART Score including a point-of-care troponin had a negative predictive value (NPV) of 98.4%.
- However, a new risk score, suitable for prehospital use, derived and validated in this study had a higher NPV and area under the receiver operating characteristic curve.
- A tailored and validated score for the evaluation of patients with chest pain to support prehospital (non-)conveyance decision-making could reduce the burden on the EMS and emergency department.

## Introduction

In western countries, chest pain represents a major reason (15% of the cases) for contact with emergency medical services (EMS).^1–3^ Its differential diagnosis is wide and ranges from non-serious musculoskeletal aetiologies to life-threatening cardiac or non-cardiac conditions. Ambulance professionals examine and treat patients on scene and decide on non-conveyance or conveyance to local or specialised hospitals. In certain situations, the decision-making process is straightforward, for example, when ST-elevation myocardial infarction (STEMI) is diagnosed on the ECG or the patient is in critical condition. However, STEMI and other obvious causes only represent a minority of cases. Decision-making on (non-)conveyance in most patients presenting with chest pain is challenging.

Consequently, a large proportion of patients presenting with chest pain are transported to the nearest emergency department (ED) to minimise mortality, severe disability and transfer accountability. However, at least 45% of the patients transported to the ED are discharged the same day without a life-threatening condition.^4^ Given the large number of patients presenting with chest pain, the current management strategy places a large burden on the healthcare system. In the Netherlands, this burden is reflected in increasing patient waiting times, frequent closures of the ED and increasing ED/EMS costs. The current management strategy might ultimately compromise overall accessibility to the healthcare system to those in need.^1–4^

For ambulance professionals, the non-conveyance decision-making process is complex and multifactorial.^5 6^ In the absence of proper support tools, (non-)conveyance is usually based on professional interpretation of signs, symptoms and electrocardiography (ECG) findings.

The History, ECG, Age, Risk Factors and Troponin (HEART) Score was developed to risk stratify patients in the ED with chest pain by their risk of having an acute coronary syndrome (ACS) and to distinguish those that may be discharged early from those that should be admitted to the hospital. The HEART Score evaluates five domains in a scale from zero to two points (the acute **H**istory of the chest pain episode, **E**CG findings, **A**ge, cardiovascular **R**isk factors and **T**roponin levels) with a possible range of zero to ten, and it stratifies patients into low (0–3), intermediate (4–7) and high risk (8–10) of ACS; see online supplemental figure S1 ^7–9^. Notably, the recent development and improvements in point-of-care (POC) troponin level measurement make it now feasible to calculate the HEART Score in the prehospital setting.^4 7 8^ Evidence for the use of this tool in the prehospital setting is lacking, and the reported incidence of 2.9% of major events in the low-risk group makes the HEART Score unsafe to rule out ACS, based on a single troponine measurement.^9–11^

10.1136/emermed-2020-210212.supp1 Supplementary data

The present study aimed to evaluate the prehospital performance of the HEART Score in patients evaluated by EMS personnel for chest pain and optimise it for the prehospital setting to conduct future prospective studies on safe (non-)conveyance decisions and management strategies.

## Methods

### Study population

Patients above 17 years of age with chest pain and a complete HEART Score, who were evaluated and transported to rule out an ACS at an ED by the EMS in the Netherlands, were eligible for the study. The northern region was considered for inclusion in our study. Those with persistent ST-segment elevation on initial ECG (ie, diagnosed with STEMI) or another apparent cause of chest pain (ie, trauma) were excluded.

Prior to the study, all EMS staff received 3 hours of education in the use of the HEART Score. In the EMS system, the HEART Score components have been systematically recorded alongside POC troponin levels since September 2016, as preparation for this study. From January 2017 to December 2017, if a patient verbally consented for participation, the EMS could calculate the HEART Score, including a troponin blood level. EMS staff was not blinded for the HEART Score nor the troponin result. Of the patients who provided written informed consent, their hospital health records were accessed. If no consent was obtained after two postal reminders, only EMS system data including vital status data (alive/deceased) were used anonymously (lacking information on ED diagnostics and discharge diagnoses). The study procedures were reviewed and approved by the local ethics committee (CardioLines 2012/296). A second (validation) cohort ran from January 2018 to July 2018, to validate the prehospital HEART (preHEART) Score. ED presentations via the EMS for chest pain were studied retrospectively in one university and two regional hospitals. The local committee waived ethical approval for this part of the study.

### POC troponin assessment

POC troponin level measurements were performed employing the dedicated system *i-STAT analyser* (Abbott Point of Care, Princeton, USA).^12^ Within the HEART Score, troponin levels were considered elevated for one point if they were above the level of detection at 0.02 μg/L and two points if they were above the 99th percentile at 0.08 μg/L in accordance with the manufacturer’s calibration study.^12^ Instructors from Abbott trained all the 255 EMS registered nurses in the use of the POC analyser. A capillary blood sampling method was developed for this study and internally validated using donated blood from 202 EMS nurses who provided informed consent. Event-time-to-troponin (ETTT) was expressed in hours between the moment of symptom onset and POC troponin level measurement, assessed by EMS staff.

### Main outcome measures

The primary endpoint of this study was major adverse cardiac events (MACEs). MACE was defined as a composite of all-cause mortality within 3 days or acute myocardial infarction (AMI) diagnosed during hospitalisation. The short timeframe was chosen to guide future (non-)conveyance decisions. Secondary endpoints were all-cause mortality at 7 and 30 days.

### Statistical analysis

Continuous variables are expressed as averages±SD or medians with IQR, as appropriate. Categorical variables are expressed as frequencies and percentages. Differences in continuous variables between groups were assessed through the Student t test, while differences in categorical variables were evaluated through Fisher’s exact test.

The performance of the HEART Score in predicting MACE was evaluated through positive and negative predictive values (PPV and NPV, respectively), and the area under the receiver operating characteristic (ROC) curves (AUC). The ability of each of the five individual components of the HEART Score was evaluated through the c-statistic. We subsequently used Youden’s J Index to optimise variable cut-offs and performed a stepwise logistic regression analysis to evaluate the significance of the optimised and available MACE predictors. Pairwise comparisons between AUCs were performed according to the method by DeLong and colleagues.^13^

Statistical significance was defined as a two-sided *p* value <0.05. All analyses were performed using SPSS V.25.0^14^ and MedCalc V.17.9.

### Patient and public involvement

The study was supported by two national patient advisory groups (*Harteraad* and *Hartpatiënten*) who participated in patient communication and study design.

## Results

**Figure 1:**
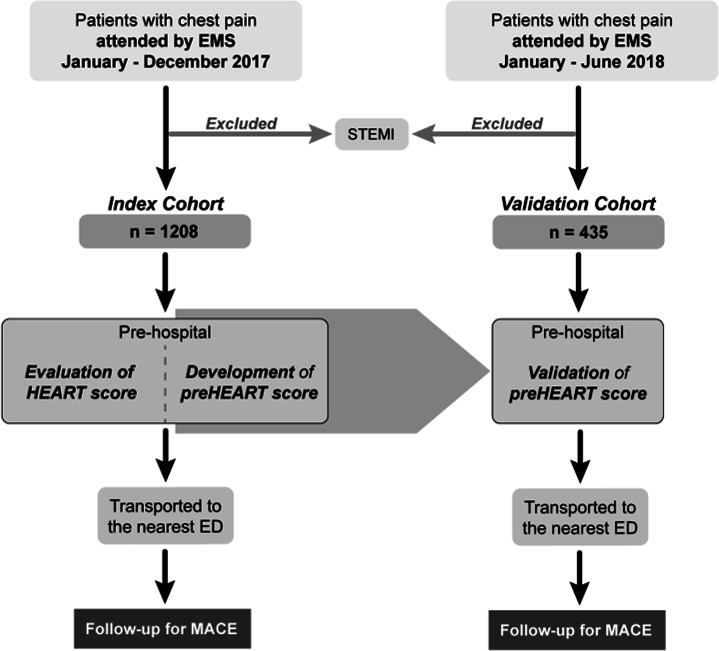
Study flow diagram. ED, emergency department; EMS, emergency medical services; HEART, history, ECG, age, risk factors and troponin; MACE, major adverse cardiac event; preHEART, prehospital HEART; STEMI, ST-elevation myocardial infarction.

Between January and December 2017, we identified 2027 patients who contacted the EMS for chest pain and were transported to an ED. One thousand two hundred eight patients gave written informed consent, and their ED and hospital data were analysed for the primary endpoint (MACE) (figure 1). Their mean age was 65.7±13.6 years, and 53% were men. Baseline characteristics of this cohort are shown in table 1. MACE within 3 days occurred in 123 (10.2%) patients (nine deaths and 114 AMIs).

**Table 1:** Baseline characteristics of the index and validation cohorts

Variables	Index cohort n=1208	Validation cohort n=435
Age, *years (SD*)	65.7±13.6	65.0±14.8
Male sex (*%*)	642 (53.1)	224 (51.5)
HR, *bpm (SD*)	91.9±19.4	94±20.3
Systolic blood pressure, *mm Hg (SD*)	154.2±29.8	153.6±27.0
Diastolic blood pressure, *mm Hg (SD*)	89.1±19.2	88.6±18.4
Event-time-to-troponin, *hours (SD*)	5.8±8.8	4.0±5.4
HEART *Score, median (IQR*)	5 (3)	5 (3)
HISTORY, *score 0 (%*)	512 (42.6)	204 (46.9)
HISTORY, *score 1 (%*)	402 (33.5)	143 (32.9)
HISTORY, *score 2 (%*)	287 (23.9)	88 (20.2)
ECG, score 0 (*%*)	522 (43.5)	202 (46.4)
ECG, *score 1 (%*)	511 (42.5)	170 (39.1)
ECG, *score 2 (%*)	168 (14.0)	63 (14.5)
AGE, *score 0 (%*)	77 (6.4)	36 (8.3)
AGE, *score 1 (%*)	441 (36.7)	164 (37.7)
AGE, *score 2 (%*)	683 (56.9)	235 (54.0)
RISK, *score 0 (%*)	163 (13.6)	46 (10.6)
RISK, *score 1 (%*)	383 (31.9)	137 (31.5)
RISK, *score 2 (%*)	665 (54.5)	252 (57.9)
TROPONIN, *score 0 (%*)	1041 (86.7)	357 (82.1)
TROPONIN, *score 1 (%*)	54 (4.5)	25 (5.7)
TROPONIN, *score 2 (%*)	106 (8.8)	53 (12.2)

HEART, history, ECG, age, risk factors and troponin; HR, heart rate.;

The secondary endpoint (all-cause mortality) was evaluated in anonymised vital status data from the entire sample (2027 patients with mean age 65.2±14.3 years and 52% men; see online supplemental table S1). Death of any cause occurred in 19 (0.9%) patients within 7 days and in 36 (1.7%) within 30 days.

### HEART Risk Score performance

The median HEART Score was five (IQR: 3–7) in the initial cohort (n=1208). The NPV, PPV and AUC were 98.4% (95% CI 96.4% to 99.3%), 35.5% (95% CI 31.8% to 39.3%) and 0.81 (95% CI 0.78 to 0.85), respectively. The proportion of patients with and without MACE across the three HEART risk categories are presented in table 2.

**Table 2:** Baseline characteristics and risk stratification of the HEART Score of patients with or without MACE and performance of the individual components of the HEART Score in the derivation cohort

	No MACE	MACE (%)	Total
**Baseline**			
Age, years *mean (SD*)	65.6 (13.6)	68.8 (12.0)	65.9 (13.5)
Male sex (*%*)	546 (50.6)	87 (75.0)	633 (53.0)
HR, *bpm (SD*)	92 (19)	91 (20)	92 (20)
Systolic BP, *mm Hg (SD*)	154 (29)	156 (30)	154 (30)
Diastolic BP, *mm Hg (SD*)	89 (19)	91 (23)	89 (19)
Event-time-to-troponin, *hours (SD*)	14 (2)	17 (4)	14 (2)
HEART *Score median (IQR*)	4 (3)	7 (3)	5 (3)
**Risk **category****			
HEART 0–3 pts	315 (98.4%)	5 (1.6%)	320 (26.5%)
HEART 4–6 pts	648 (92.7%)	51 (7.3%)	699 (57.9%)
HEART 7–10 pts	122 (64.6%)	67 (35.4%)	189 (15.6%)
**Performance**	**AUC**	**95% CIs**	**P value**
HEART Score	0.814	0.78 to 0.85	0.00
**Components**			
History	0.693	0.65 to 0.74	0.00
ECG findings	0.686	0.63 to 0.74	0.00
Age	0.560	0.51 to 0.61	0.03
Risk factors	0.551	0.50 to 0.60	0.06
Troponin	0.714	0.66 to 0.77	0.00

AUC, area under the receiver operating characteristic curve; BP, blood pressure; HEART, history, ECG, age, risk factors and troponin; HR, heart rate; MACE, major adverse cardiac event.;

### Optimisation of the HEART Risk Score for prehospital application

To refine and optimise the HEART Score for use in the prehospital setting, we analysed the performance of its individual components for MACE using the AUC (table 2). Three components showed significant discrimination between MACE and no MACE: h*istory* (p<0.01), *ECG* findings (p<0.01) and *troponin* levels (p<0.01). *Age* provided significant but limited discrimination of AUC 0.560 (p=0.03). However *risk* (cardiovascular risk factors) demonstrated non-significant discrimination (p=0.06).

We used Youden’s J Index to adjust thresholds for optimising age groups (due to the limited discrimination performance with the current cut-offs). The optimised thresholds for age were 18–40, 40–70 and >70 years old (c=0.61, p<0.001), Similarly, a new 99th threshold for elevated troponin value was calibrated (as the prehospital setting of this study implied an earlier measurement than in studies taking place in the ED). The optimised values were ≤0.02 μg/L, 0.03–0.04 μg/L and ≥0.05 μg/L (c=0.73, p<0.001).

**Figure 2:**
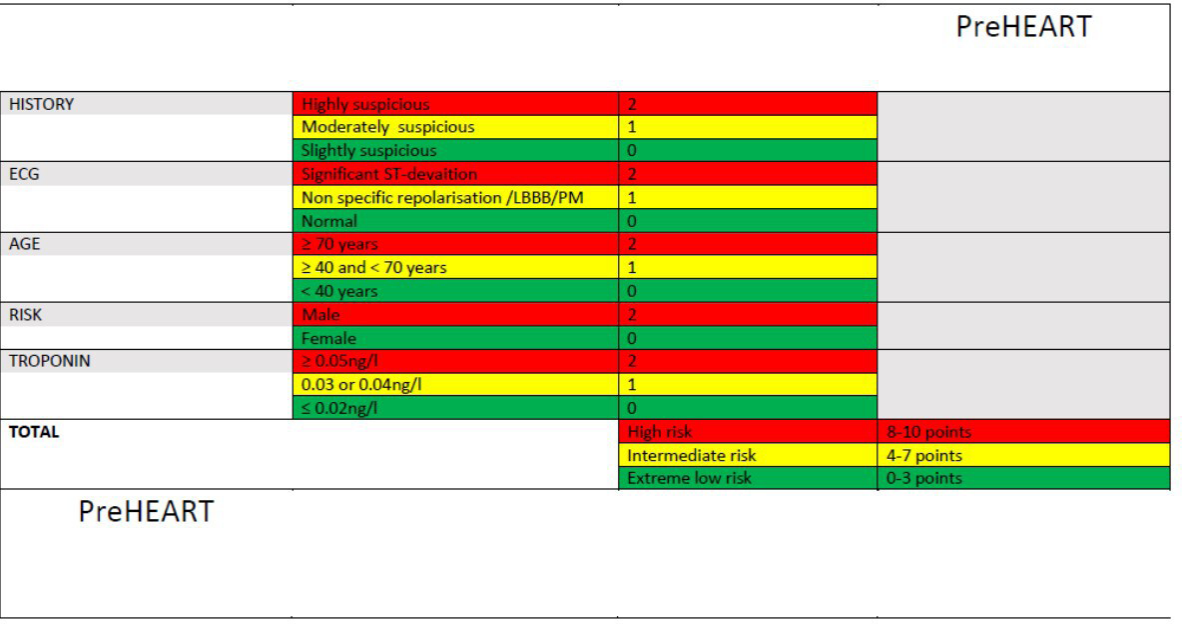
The prehospital History, ECG, Age, Risk Factors and Troponin (preHEART) Risk Score. The five domains are assigned 0–2 points with a total score ranging from 0 to 10. Patients are then stratified into an extreme low (0–3 points), intermediate (4–7) or high (8–10) risk of major adverse cardiac events category. Abbreviations: LBBB, Left Bundle Branch Block, PM, Pacemaker

A stepwise logistic regression analysis was performed, including the four significant and optimised HEART Score variables and EMS-available predictors (sex, heart rate and systolic and diastolic blood pressure). *History*, *ECG* findings, age, *troponin* levels and male sex (as a single risk factor) were independent predictors of MACE (see online supplemental table S2). These variables were considered together for a modified version of the HEART Score optimised for prehospital application (*preHEART Risk Score*). The risk groups derived from the preHEART Score were low (0–3 points), intermediate (4–7 points) and high (8–10 points) to further optimise NPV and PPV; see figure 2.

The preHEART Score in this derivation cohort performance had an NPV of 99.3% (95% CI 98.07 to 99.78), a PPV of 49.4% (95% CI 41.96 to 56.86) and an AUC of 0.85 (95% CI 0.82 to 0.88). Comparatively, the preHEART Score outperformed the HEART Score (p=0.01) and troponin levels alone (the strongest single MACE predictor overall) (p<0.01), while the HEART Score showed comparable performance to troponin levels alone in the prediction of MACE. The ROC curve analyses are depicted in online supplemental figure S2. In this cohort, the median preHEART Score was four (IQR: 3–7). The proportion of patients with and without MACE across the preHEART risk categories are presented in online supplemental table S3.

Performance results considering the secondary endpoints (all-cause death at 7 and 30 days and ETTT threshold) showed similar results, shown in table 3 and online supplemental table S4.

**Table 3:** Event-time-to-troponin and MACE, threshold with AUC, 95% CI, negative and positive predictive values (NPV and PPV) of the original HEART Score versus the preHEART Score of the index cohort and DeLong test for significance difference of non-parametric ROC curves. n=1208

ETTT/%	HEART				preHEART				HEART versus preHEART
	MACE NPV	MACE PPV	ROC	95% CI	MACE NPV	MACE PPV	ROC	95% CI	P value
0 hours/100	98.5%	35.4%	0.81	0.78 to 0.83	99.3%	49.4%	0.85	0.82 to 0.89	0.01
1 hour/78.0	98.1%	32.8%	0.80	0.75 to 0.85	99.0%	49.2%	0.85	0.81 to 0.89	0.07
2 hours/59.6	98.1%	33.0%	0.82	0.77 to 0.88	99.2%	51.0%	0.86	0.82 to 0.91	0.35
3 hours/50.6	97.7%	31.1%	0.81	0.75 to 0.88	99.0%	54.8%	0.86	0.81 to 0.91	0.15
4 hours/43.3	98.2%	32.4%	0.82	0.75 to 0.88	98.9%	51.4%	0.86	0.81 to 0.92	0.32
5 hours/40.0	98.0%	30.3%	0.81	0.74 to 0.89	98.7%	50.0%	0.86	0.79 to 0.92	0.66

AUC, area under the ROC curves; ETTT, event time to troponine; HEART, history, ECG, age, risk factors and troponin; MACE, major adverse cardiac event; NPV, negative predictive value; PPV, positive predictive value; preHEART, prehospital HEART; ROC, receiver operating characteristic.

### Validation of the preHEART Score

To validate the preHEART Score, we analysed the second consecutive cohort (recruited between January and June of 2018). This validation cohort consisted of 435 patients who presented to the ED for chest pain via the EMS. The average age was 65±14.8 years, and 52% of patients were men (table 1). A MACE occurred in 53 patients (12.2%), while at 7 and 30 days, two (0.5%) and seven (1.6%) patients died. The distribution of patients across the preHEART risk categories is shown in online supplemental table S5. In this validation cohort, the preHEART Score again performed better than the HEART Score with an NPV, a PPV and an AUC of 99.4% (95% CI 96.0 to 99.9), 50.0% (95% CI 37.3 to 62.7) and 0.84 (95% CI 0.79 to 0.88), respectively.

## Discussion

The present study evaluated the prehospital performance of the HEART Score in predicting MACE, as applied by EMS personnel in patients presenting with chest pain, and included the use of POC troponin measurement. Furthermore, the HEART Score’s components were evaluated and adjusted to develop the preHEART Score, a decision support tool optimised for application in the prehospital setting. Finally, a second independent cohort validated the preHEART Score.

### Prehospital risk assessment performance

There is a general need to optimise the prehospital evaluation of patients with chest pain, and the use of dedicated risk scores to support decisions is likely to improve the quality of (non-)conveyance decisions.

The integration of relevant and easy-to-evaluate variables in the HEART Score offers the possibility to perform further care decisions in patients evaluated by EMS due to chest pain. Current state-of-the-art POC troponin measurements make it possible to incorporate this powerful marker of myocardial damage into a useful and systematic tool such as the HEART (and preHEART) Score, as demonstrated by the present study.

Our results confirmed that the HEART Score’s main strength is found in its capacity to identify low-risk patients as attested by its high NPV (98.4%), while its PPV is rather limited (35.4%). In the prehospital setting, however, misclassifications are quite relevant. Furthermore, considering that the HEART Score was initially developed for use in the ED, we deemed that evaluating and possibly optimising it for prehospital use by EMS personnel are important. The examination of the discriminative performance of the individual components in the HEART Score revealed two until now unrecognised aspects: first, that the ‘risk factors’ variable does not seem to significantly contribute to the discrimination capacity of the HEART Score for MACE in the prehospital setting and, second, that further optimisation of thresholds for age and troponin levels could in fact improve the score’s performance. In terms of the former, the evaluation of risk factors is operationally difficult due to the lack of complete health records in the on-scene ambulance scenario^15–17^ (in contrast with the extensive records that can be consulted in the ED by attending physicians) as well as the time-sensitive and pressing nature of the EMS contact. We speculate that suboptimal scoring of cardiovascular risk factors and symptoms in female patients may have partially explained the lack of significance of this component. Previous research has additionally suggested that the HEART Score may perform differently in men and women. Therefore, the prehospital optimised structure of the HEART Score (abbreviated to the *preHEART* Score) replaces ‘risk factors’ for male sex (as a statistically relevant predictor), improving the overall performance of the score.

Previous work by van Dongen and colleagues has evaluated the modified HEART Score in the EMS setting. They did not investigate the performance of its individual components in the prehospital field.^15^ Notably, their report documented 2.9% of MACE in the low-risk group, which would be inadequate in the prehospital risk assessment. Moreover, previous studies have not focused on high-risk patients. Conversely, our study reports that optimisation of the score’s risk levels (into the preHEART Score) also improves the PPV. Only three of the eight available hospitals have percutaneous coronary intervention (PCI) facilities in our healthcare region. Hence, there is additional value in better identification of patients at high risk of requiring interventional treatment, with a promise of earlier treatment of ACS, prevention of double diagnostics and less interhospital transport of patients

These insights should encourage the implementation of robust prehospital risk stratification of patients with chest pain. This is of major interest because the optimised preHEART Score justifies the exploration of effectiveness in determining group-specific care paths. Currently, approximately 25% of patients presenting with chest pain are left at home based on EMS personnel’s professional assessment. This decision of (non-)conveyance is considered difficult, and decision support tools might optimise this partly subjective assessment. For example, the preHEART Score could be added as a decision support tool to identify low-risk patients and discuss the option to remain at home, avoiding unnecessary transportation, admission to the ED and non-elective procedures. Also, an improved stratification of the high-risk patients with potentially ongoing non-STEMI (NSTEMI) may enhance their early identification, and treatment paths might be further optimised in the way that current care for STEMI patients is performed (ie, direct transport to a PCI facility).

### False-negative classifications

A naturally arising concern is patient safety, especially when the risk score indicates non-conveyance. However, when closely looking at the patients in this study who experienced an event while considered as low risk, we deem its application as safe, as the harm in these cases did not appear to be substantial. In the derivation cohort, the first case was an 80+-year-old patient, diagnosed with atrial fibrillation and registered as NSTEMI but with a documented troponin peak of 0.02 μg/L, suggesting a misclassification. The second was an 85+-year-old patient presenting with a supraventricular tachycardia who was also registered with NSTEMI but with an ED troponin level of 0.04 μg/L. This patient directly refused further diagnostics or treatment. A third case was an 80+-year-old patient with acute leukaemia who died after 2 days from related complications. Furthermore, in the validation cohort, only one 45+-year-old patient with renal failure suffered an NSTEMI (registered peak troponin of 0.04 μg/L) and was treated conservatively.

### Future perspectives

Further research should focus on developing management pathways to implement the preHEART Score as a decision support tool. Correct stratification can avoid unnecessary transport of a low-risk patient, while improving the transportation indication for patients at high preHEART risk could also be considered. Other future developments in the prehospital setting may include further POC biomarkers (eg, D-dimer and natriuretic peptides) to expand the risk stratification and diagnostic capabilities on scene.

### Limitations

Several limitations of this study should be considered. Within the validation cohort, MACE was defined based on hospital discharge documentation and conclusion, not further interpreted by the research or adjudicated by an event committee. Furthermore, in the cases deemed as low risk that presented an event, there were also additional reasons to justify the ED’s conveyance. It is possible that the preHEART Score’s performance may have been underestimated and that the NPV will possibly reinforce the notion of safety on non-conveyance in the future.

The HEART Risk Score performs reasonably well for stratification of patients with chest pain according to their risk of MACE; however, the preHEART preforms better in the prehospital setting. The preHEART Score seems to be particularly useful in identifying patients at very low risk as opposed to the (modified) HEART Score. Further prospective research into the use of the preHEART Score for (non-)conveyance decisions in patients with chest pain is recommended.

## Data Availability

Data are available on reasonable request. All deidentified data can and will be shared on a written reasonable request.
