# Supplementary material for: Prehospital risk stratification in patients with chest pain
Source: Emerg Med J. 2021 Aug 9;38(11):814–9. doi: 10.1136/emermed-2020-210212 (PMC8551969; doi:10.1136/emermed-2020-210212)
Supplement: Supplementary data [file emermed-2020-210212supp001.pdf]

BMJ Publishing Group Limited (BMJ) disclaims all liability and responsibility arising from any reliance placed on this supplemental material which has been supplied by the author(s)

Pre-hospital risk stratification in patients with chest pain

ONLINE SUPPLEMENTARY MATERIAL

Figure S1 The HEART score

HEART

HEART score for chest pain patients			
History	Highly suspicious	2	
	Moderately suspicious	1	
	Slightly suspicious	0	
ECG	Significant ST-deviation	2	
	Non specific repolarisation disturbance / LBTB / PM	1	
	Normal	0	
Age	≥ 65 years	2	
	> 45 and < 65 years	1	
	≤ 45 years	0	
Risk factors	≥ 3 risk factors or history of atherosclerotic disease*	2	
	1 or 2 risk factors	1	
	No risk factors known	0	
Troponin	≥ 3x normal limit	2	
	> 1 and < 3x normal limit	1	
	≤ 1x normal limit	0	
		Total	

***Risk factors for atherosclerotic disease:**

Hypercholesterolemia
Hypertension
Diabetes Mellitus
Cigarette smoking
Positive family history
Obesity

Table S1. Baseline characteristics of the 2027 patients with all-cause mortality.

	**N = 2027**	
**Variable**		
Age *years (SD)*	65.2 ± 14.3	
Male sex *(%)*	1057 (52.1)	
HR bpm *(SD)*	92 ± 19	
Systolic blood pressure *mmhg (SD)*	154.7± 29.8	
Diastolic blood pressure *mmhg (SD)*	89.3 ± 18.9	
Event-time-to-troponin *hrs. (SD)*	3.8 ± 5.2	
HEART *score median (IQR)*	4 (3)	
HISTORY *score 0 (%)*	928 (45.0)	
HISTORY *score 1 (%)*	667 (32.4)	
HISTORY *score 2 (%)*	465 (22.6)	
ECG *score 0 (%)*	924 (44.9)	
ECG *score 1 (%)*	855 (41.5)	
ECG *score 2 (%)*	281 (13.6)	
AGE *score 0 (%)*	159 (7.7)	
AGE *score 1 (%)*	773 (37.5)	
AGE *score 2 (%)*	1128 (54.8)	
RISK *score 0 (%)*	273 (13.3)	
RISK *score 1 (%)*	674 (32.7)	
RISK *score 2 (%)*	1113 (54.0)	

Abbreviations: *BP, blood pressure; HR, heart rate;*

Figure S2:

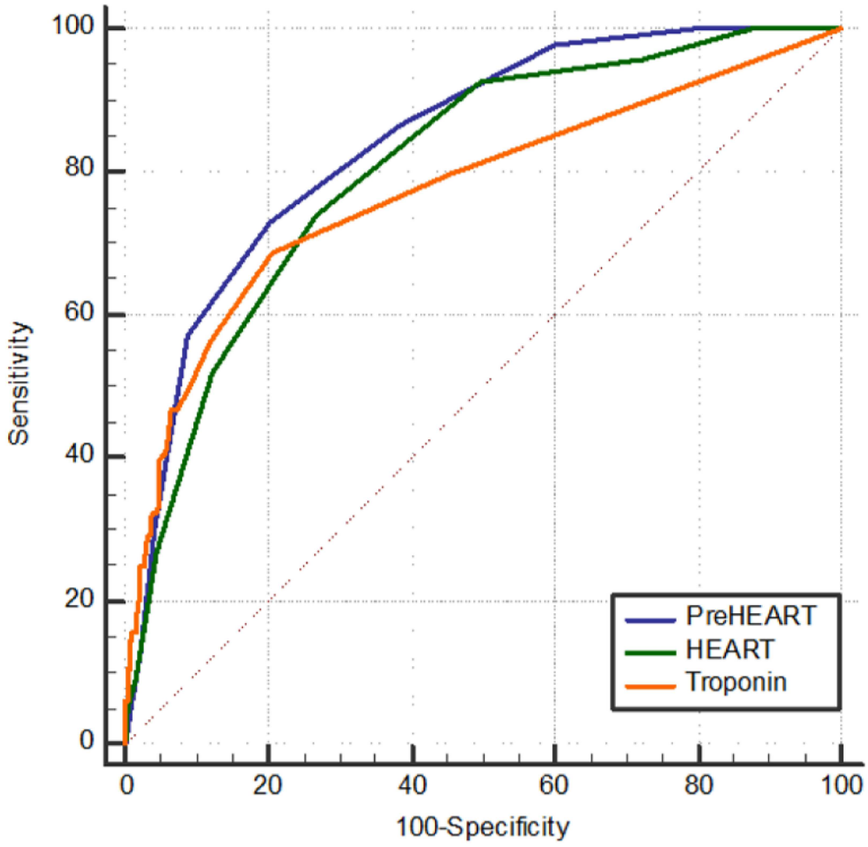
100
80
60
40
20
0
Sensitivity
0
20
40
60
80
100
100-Specificity
PreHEART
HEART
Troponin

Table S2. PreHEART score development

	OR	*95%CIs*	*p-value*	*OR*	*95%CIs*	*p-value*
Variables						
History	*2.08*	*1.46-2.97*	*0.00*	*2.02*	*1.52-2.69*	*0.00*
ECG	*1.32*	*0.91-1.92*	*0.15*	*1.57*	*1.15-2.14*	*0.00*
Age (optimized)	*1.98*	*0.90-4.34*	*0.09*	*2.33*	*1.54-3.54*	*0.00*
Troponin (optimized)	*2.68*	*1.24-5.79*	*0.01*	*2.80*	*1.26-3.53*	*0.00*
Male sex	*1.51*	*1.12-2.05*	*0.01*	*1.59*	*1.26*	*0.00*
Heart rate	*0.99*	*0.97-1.00*	*0.12*	..	..	..
Systolic BP	*1.00*	*0.99-1.02*	*0.68*	..	..	..
Diastolic BP	*1.01*	*0.99-1.03*	*0.56*	..	..	..
Event-to-troponin-time	*1.00*	*1.00-1.00*	*0.77*	..	..	..

Abbreviations: *OR, odds ratio; CIs, confidence intervals; BP, blood pressure*

Table S3. Risk stratification and performance of the optimized preHEART score

	No MACE	***MACE***	***Total***
Risk category			
Low risk (n)	455 (99.3%)	3 (0.7%)	458 (37.9%)
Intermediate risk (n)	588 (88.2%)	79 (11.8%)	667 (55.2%)
High risk (n)	42 (50.6%)	41 (49.4%)	83 (6.9%)
Performance	***AUC***	***95% CIs***	***p-value***
PreHEART score	*0.85*	*0.82-0.88*	*<0.01*

Abbreviations: *AUC, area under the curve; MACE, major adverse cardiac events; CIs, confidence intervals*

Table S4. Performance (in AUCs) of the HEART and preHEART scores considering the secondary endpoints, mortality at 7 and 30 days after first medical contact.

**Death at 7 days**	**Index Cohort**	**CI 95%**	**Validation Cohort**	**CI 95%**
**HEART**	0.81	0.79-0.83	0.81	0.77-0.85
**preHEART**	0.85	0.85-0.87	0.83	0.79-0.87
**Death at 30 days**	**Index Cohort**	**CI 95%**	**Validation Cohort**	
**HEART**	0.80	0.78-0.82	0.82	0.78-0.85
**preHEART**	0.84	0.82-0.86	0.84	0.80-0.87

***Abbreviations**: CI, confidence interval.*

Table S5 Distribution of patients across the HEART and preHEART scores in the index and validation cohorts

	**Index Cohort**<br>N=1208	**Validation Cohort**<br>N=435
**HEART** score, median (IQR)	5 (3)	5 (3)
HEART low-risk (%)	320 (26.5)	123 (28.3)
HEART intermediate-risk (%)	699 (57.9)	234 (53.8)
HEART high-risk (%)	189 (15.6)	78 (17.9)
**preHEART** score, median (IQR)	4 (2)	4 (2)
preHEART low-risk (%)	458 (37.9)	160 (36.8)
preHEART intermediate-risk (%)	668 (55.3)	247 (56.8)
preHEART high-risk (%)	82 (6.8)	28 (6.4)

***Abbreviations**: IQR: interquartile range*
